# Collaborating with Virtual Assistants in Organizations: Analyzing Social Loafing Tendencies and Responsibility Attribution

**DOI:** 10.1007/s10796-021-10201-0

**Published:** 2021-10-21

**Authors:** Stefan Stieglitz, Milad Mirbabaie, Nicholas R. J. Möllmann, Jannik Rzyski

**Affiliations:** 1 Digital Communication and Transformation, University of Duisburg-Essen, Duisburg, Germany; 2 Faculty of Business Administration and Economics, Paderborn University, Paderborn, Germany

**Keywords:** Virtual collaboration, Virtual assistants, Social loafing, Responsibility attribution, Organizations

## Abstract

Organizations increasingly introduce collaborative technologies in form of virtual assistants (VAs) to save valuable resources, especially when employees are assisted with work-related tasks. However, the effect of VAs on virtual teams and collaboration remains uncertain, particularly whether employees show social loafing (SL) tendencies, i.e., applying less effort for collective tasks compared to working alone. While extant research indicates that VAs collaboratively working in teams exert greater results, less is known about SL in virtual collaboration and how responsibility attribution alters. An online experiment with N = 102 was conducted in which participants were assisted by a VA in solving a task. The results indicate SL tendencies in virtual collaboration with VAs and that participants tend to cede responsibility to the VA. This study makes a first foray and extends the information systems (IS) literature by analyzing SL and responsibility attribution thus updates our knowledge on virtual collaboration with VAs.

## Introduction

In today’s business world, technological advancements constantly reshape organizational efforts for remaining competitive (Cetindamar Kozanoglu & Abedin, 2020; N. Frick & Marx, 2021; Soto Setzke et al., 2021), transforming digital workplaces in enterprises for exploiting relative advantages (Junglas et al., 2019; Majhi et al., 2021; Meske & Junglas, 2020). This also implies employees and teams increasingly collaborate with and via technology (Changizi & Lanz, 2019; de Vreede & Briggs, 2005). The collaboration with technologies enabled by artificial intelligence (AI), such as virtual assistants (VAs), shifts from simply using a tool for virtual collaboration with other employees to shaping a novel and independent virtual environment to collaborate with VAs (Maedche et al., 2019; Mirbabaie et al., 2020; Seeber, Waizenegger, et al. 2020a). VAs are software dialog systems simulating the behavior of humans which can be addressed via voice- or text-based commands and respond to the users’ input appropriately (Brachten et al., 2020; Mirbabaie et al., 2020, 2021b). The application possibilities in organizations are manifold but VAs are foremost used as work facilitators (Brachten et al., 2020; Luger & Sellen, 2016; Mirbabaie et al., 2020). VAs collaborate with employees to optimize internal processes (Norman, 2017), generate additional revenue or cost savings (Quarteroni, 2018), and increase customer satisfaction (Behera et al., 2021; Verhagen et al., 2014), thus aim to establish substantial advantages over market competitors (Benbya & Leidner, 2018; Yan et al., 2018). Even though VAs do not provide a physical interaction and human representation (Maniscalco et al., 2020), they are increasingly used in virtual collaboration (Panganiban et al., 2020; Seeber et al. 2020a) and their distribution in enterprises is likely to grow (Maedche et al., 2019).

Many teams in organizations collaborate in virtual teams, which might even be globally distributed (Andres & Shipps, 2019; Hassell & Cotton, 2017; Massey et al., 2003; Plotnick et al., 2016). Virtual collaboration happens through using simple tools such as Microsoft Teams or Slack (N. R. J. Frick, et al., 2021a, 2021b, 2021c) and reaches to computer-generated virtual realities (Fromm et al., 2020; Litvinova et al., 2018). Existing research takes the standpoint that employees and VAs collaboratively working in virtual teams exert greater results (Seeber, et al., 2020b; Waizenegger et al., 2020). However, less research is concerned with potential downsides. As in virtual collaboration, working with members in physical teams should inspire individuals to maximize their potential and to work particularly hard (Harkins & Petty, 1982). Nevertheless, research on teamwork also identified social loafing (SL), i.e., individuals working less for collective tasks than for individual tasks (Hardy & Latané, 1988; Karau & Williams, 1993). Employees might apply less effort to achieve a goal in a team compared to when working alone as the individual contribution is perceived as unnecessary and/or responsibility attribution is distributed among team members (Jassawalla et al., 2009; Karau & Williams, 1993; Latané et al., 1979). In corporate contexts, a possible decrease in motivation is one of the biggest obstacles for teamwork (George, 1992); it is thus vital to minimize individual tendencies to loaf (Schippers, 2014) especially for maintaining cohesiveness of teams (Taylor et al., 1983).

SL has been observed multiple times in physical teams uncovering scientific evidence that individuals working in teams show less effort compared to working independently (Karau & Williams, 1993; Latané et al., 1979). However, individuals increasingly collaborate in virtual teams with VAs (Maedche et al., 2019; Mirbabaie et al., 2020; Seeber, et al., 2020a, 2020b, 2020c) thus scholars need to adjust their understanding on the different aspects of collaborative settings (Mirbabaie et al., 2020). To gain a deeper understanding on virtual collaboration with VAs, knowledge from human-to-human collaboration research needs to be exploited (Demir et al., 2020). As collaboration with VAs in organizations is likely to become commonplace, and VAs are increasingly perceived as human-like actors since they are being inherently anthropomorphic (Feine et al., 2019; Hussain et al., 2019; Pfeuffer et al., 2019; Porra et al., 2020), there is an urgent demand for the information systems (IS) discipline to conduct further research on virtual collaboration to reveal differences and similarities to human teams (Mirbabaie et al., 2021a). Associated consequences might significantly alter theoretical and practical viewpoints of how and for what purpose VAs are applied in virtual collaboration. Employees no longer identifying themselves with a decision made by a VA, not questioning or taking responsibility for it, which possibly means making poor or even wrong choices (Trocin et al., 2021). This generates enormous risks for both enterprises (i.e., diminishing reputation or profitability) and individuals (i.e., feeling less valued and more stressed) (Chidambaram & Tung, 2005; Grimes et al., 2021). Missing responsibility attribution in human-VA teams decisively thus impacts the way VAs are implemented and how they are being applied in organizations, restricting their deployment to certain industries, departments, teams and purposes. Moreno et al. (2001) clarified that there is generally a contradiction on positive (constructivist hypothesis) and negative (interference hypothesis) outcomes when collaborating with VAs. On the one hand, VAs assist employees in virtual collaboration, freeing them from unwanted duties and allowing them to focus on their key responsibilities (Brachten et al., 2020). On the other hand, intensified collaboration with VAs might yield in employees becoming dependent and showing greater SL tendencies and missing responsibility attribution (Mirbabaie et al., 2021b). To examine the role of VAs in virtual collaboration and related consequences regarding SL and responsibility attribution, our research is guided by the following questions:

### RQ1

To what extent do virtual assistants cause social loafing in virtual collaboration?

### RQ2

How does responsibility attribution differ in virtual teams?

To answer these questions, we conducted an online experiment with 102 participants who were assisted in the execution of a work-related task by a text-based VA. We measured and compared general SL behavior and SL behavior in virtual collaboration with a VA as well as the attribution of responsibility. This study contributes to theory and practice by structuring our understanding of collaboration with VAs and related negative effects. Researchers will find the novel insights fruitful in understanding what consequences are related to virtual collaboration with VAs assisting in work-related tasks. Practitioners, such as managers and software developers, will be able to comprehend possible downsides for human-VA teams and which measures might be rewarding to cope and/or prevent negative outcomes. This article seeks to extend the IS literature by attempting to make a first foray into the examination SL tendencies and responsibility attribution in virtual collaboration with VAs to drive future research in this field of high relevance.

## Related Work

**Fig. 1:**
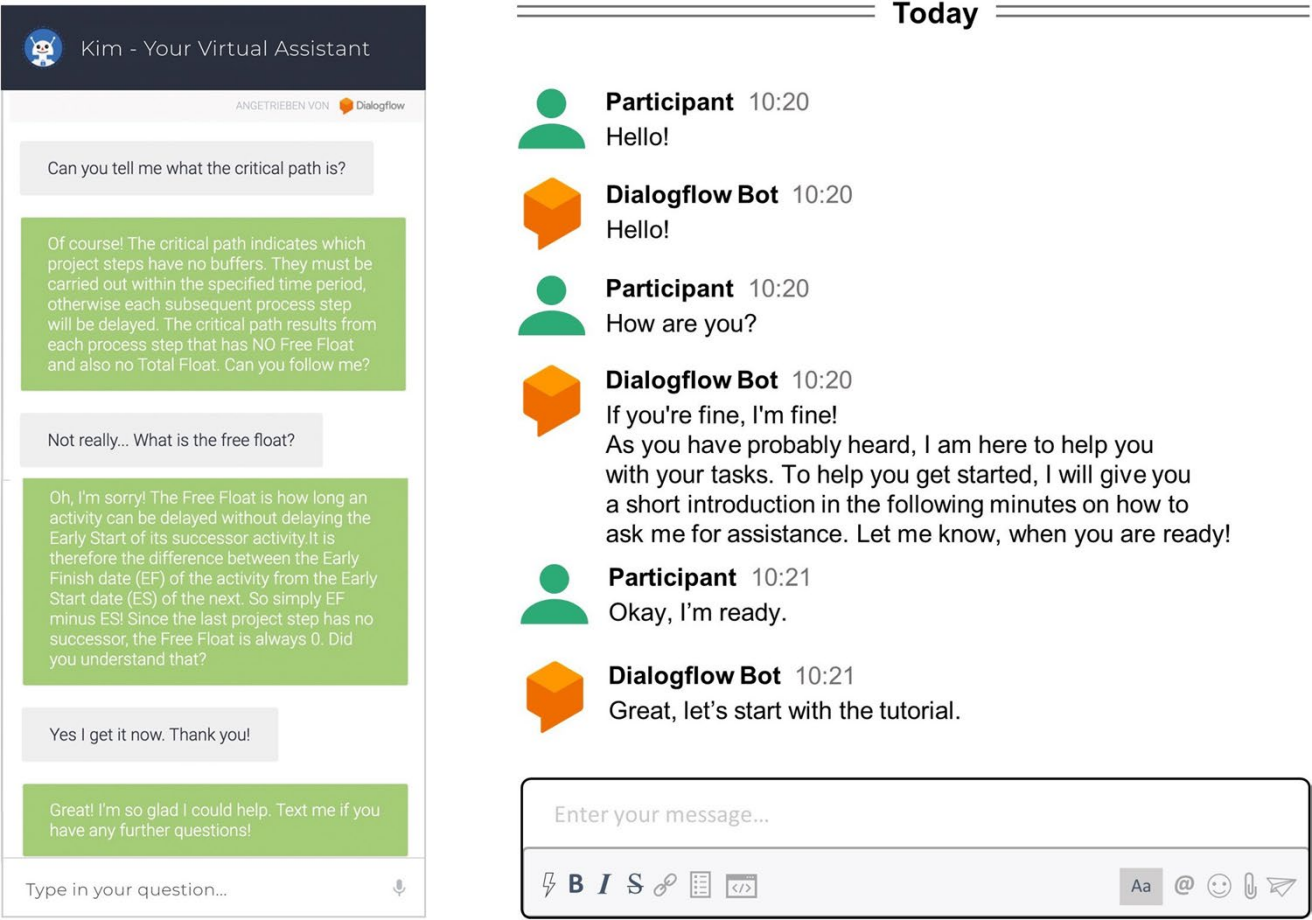
Example interactions with VAs from earlier research (Brachten et al., 2020; Mirbabaie et al., 2020)

Collaboration with technology was examined from multiple perspectives within the IS discipline (Bajwa et al., 2007; Bednar & Welch, 2020; Beer et al., 2005; Frohberg & Schwabe, 2006; Schwabe, 2003; You & Robert, 2018). Throughout decades of fundamental research, several terms evolved for systems behaving alike. Popular terms that are related to VAs and found in theory and practice are chatbots (Stieglitz et al., 2018), digital assistants (Maedche et al., 2019) and conversational agents (Frick et al., 2021a, 2021b, 2021c). Scholars examined VAs from different perspectives (e.g., Luger & Sellen, 2016; Saffarizadeh et al. 2017; Seeber et al., 2018). Gnewuch et al. (2017) differentiated VAs in their primary mode of communication (how users interact with VAs) and their main purpose (whether a VA covers a narrow or broad task). Knote et al. (2019) characterized VAs by their design characteristics, for instance, adaptivity (how VAs learn and adjust to changing environments) and assistance domain (in which area VAs provide assistance). While research is lacking a consistent classification of VAs due to possible overlaps in their capabilities (Mirbabaie et al., 2021b), multiple definitions emerged but somewhat limit the manifold functionalities of VAs. In this research we follow the interpretation of Mirbabaie et al. (2021b) who defined VAs as “dialog systems simulating the behavior of humans via different modes of communication (e.g., written or spoken natural language, haptics, gestures, facial expressions, graphics), continuously learn and develop over time by analyzing and interpreting a given input combined with additional data sources for assisting with divergent tasks or execute them autonomously” (p. 4). Figure 1 outlines two examples of VAs that were used in earlier studies (Brachten et al., 2020; Mirbabaie et al., 2020).

VAs have become increasingly relevant as work-facilitator in organizations in recent years due to constant technological enhancements (Berg, 2015; Spohrer & Banavar, 2015) turning regular information technology (IT) or decision support systems into smart systems (Alter, 2020). The development of VAs is based on AI and underlying machine learning (ML) algorithms attempting to create intelligent systems augmenting the abilities of humans (McTear, 2017). However, there are certain distinctions of intelligent behavior (Mirbabaie et al., 2020). One the one hand, there are agents that respond to a certain input based on predefined rules (Russel & Norvig, 2016). On the other hand, there are adaptive systems that behave reactively, proactively and autonomously (von Wolff et al., 2019) while continuously learning and developing over time by processing different types of digital information (Mitchell et al., 2018). VAs aim to combine the complementary strengths of humans and AI (Kamar, 2016), for example, associating emotional intelligence of humans with the processing of huge amounts of data (Dellermann, et al., 2019a, 2019b). Thereby, VAs supply humans with feedback to assist in decision-making (e.g., AI in the loop of human intelligence), and humans supply VAs with feedback to optimize their capabilities (e.g., human intelligence in the loop of AI) (Dellermann, et al., 2019a, 2019b; Dellermann, et al., 2019a, 2019b; Mirbabaie et al., 2021b).

In virtual collaboration, VAs use multiple communication styles, such as written or spoken natural language, and are capable to interpret and react to gestures or facial expressions (Laumer et al., 2019; Nakano & Fukuhara, 2012). They adapt to users with varying roles by addressing them via different languages (Gnewuch et al., 2020; Pfeuffer et al., 2019), interpret emotions of individual team members (McDuff & Czerwinski, 2018) and foster disclosing of relevant information (Frick et al., 2021a, 2021b, 2021c). VAs are further inherently anthropomorphic since they use verbal, visual, auditory, and invisible social cues, for instance jokes, facial expression, laughing and response time, which makes users feel like talking to another human (Feine et al., 2019; Hussain et al., 2019; Pfeuffer et al., 2019). Within the research stream of CASA (Computers Are Social Actors) it has been identified that individuals exhibit social reactions when collaborating with technology (Nass & Moon, 2000). As explained by the Social Response Theory, individuals subconsciously associate social rules towards technology that uses human-like traits or behavior (Nass & Moon, 2000; Reeves & Nass, 1996). Furthermore, individuals have certain expectations towards VAs that use human-like design characteristics which might not be in line with their actual capabilities or purpose (Luger & Sellen, 2016). Nevertheless, yet existing limited conversational skills of VAs remind individuals that they are still collaborating with a machine, raising feelings of strangeness possibly leading to a discontinued usage (Diederich et al., 2020). Furthermore, there are also certain challenges and dangers related to security and privacy heavily impacting trust in VAs (de Barcelos Silva et al., 2020; Lee et al., 2020). For example, the feeling of being surveilled or that data is misused or retrieved for dubious purposes decreases trust in the agent and hinders the distribution of information (Frick et al. 2021). Moreover, interacting with VAs increases the transparency of working methods, roles and tasks which causes discomfort (Wünderlich & Paluch, 2017) and yields in a feeling of being monitored, losing personal value and control as well individual knowledge (Mirbabaie et al., 2020).

The application of VAs in organizational surroundings is fruitful on many levels and is already implemented across industries. VAs are used internally and externally to optimize processes by assisting in the execution of work-related tasks (Norman, 2017), increase the overall customer satisfaction (Cui et al., 2017; Verhagen et al., 2014), establish substantial competitive advantage (Benbya & Leidner, 2018) and generate additional revenue or cost savings (Quarteroni, 2018). For example, VAs are used in form of a virtual colleague from the human resource department to enhance the onboarding processes of new hires by providing flawless guidance (Shamekhi et al., 2018). Furthermore, VAs are applied in customer service and assist in answering customer inquiries more quickly (Gnewuch et al., 2017; Hu et al., 2018). In principle, the application of VA in organizations aims to reduce the workload of employees by assisting with repetitive tasks (Brachten et al., 2020; McTear, 2017; Mirbabaie et al., 2020).

Recent research demonstrates that VAs are able to assist in the decision-making process (Seeber, et al., 2020b; Waizenegger et al., 2020) and tackle collaborative issues frequently arise (Mirbabaie et al., 2021b). However, since VAs are becoming more human-like (Feine et al., 2019; Hussain et al., 2019; Pfeuffer et al., 2019) and are already perceived as legitimate team members (Seeber et al., 2018) it can be assumed that knowledge from human teams is transferable to virtual collaboration with VAs (Mirbabaie et al., 2020). This includes positive and negative aspects and possibly disadvantages known from human–human collaboration.

## Theoretical Background

### The Phenomenon Social Loafing

In 1913, Maximilien Ringelmann conducted an experiment to suggest a possible decrement in the individual motivation as a result of working in a group (Kravitz & Martin, 1986). Male volunteers were asked to pull on a rope as hard as they can (tug-of-war) in groups of varying sizes where a measuring device determined the total effort. The results showed that as the group sizes increased, the group performance was increasingly lower than from the addition of individual performances. This behavior was later explained as SL, “the reduction in motivation and effort when individuals work collectively compared with when they work individually” (Karau & Williams, 1993, p. 681). SL might arise when individuals perceive their contribution to be unnecessary and/or responsibility is distributed among members of a team (Karau & Williams, 1993; Latané et al., 1979). SL is also expected to rise when team sizes increase as the responsibility is distributed among several humans (Dennis et al., 2005). A decrease of SL is possible when individuals believe that they are being monitored and measured by their personal performance (Karau & Williams, 1993). Thus, the individual contribution is reduced when members of nominal groups believe that they are collectively working in teams.

While the observability of SL is hardly contestable, there is still no clear explanation for this social phenomenon. An extensive body of literature focused on the different determinants (Vaghefi & Lapointe, 2012), such as individual characteristics and differences, group sizes and memberships or task contribution efforts and visibility (e.g., Gavala-González et al., 2020; ONeill et al., 2020; Zhu et al., 2019). For example, Smith et al. (2001) observed that a lower need for cognition is associated with SL. Individuals who enjoy and participate in elaborating cognitive tasks are less likely to reduce their efforts in groups. Schippers (2014) examined personality traits and indicated that a high occurrence of conscientiousness and agreeableness within a group might compensate SL tendencies. In contrast, a recent study of Hou et al. (2021) examined SL in online brand communities and revealed that agreeableness and conscientiousness do not have a direct impact on SL. Nevertheless, the authors explained that personality traits are indirectly related to SL behavior. Research further identified apathy and social disconnectedness as antecedents for SL and explained that participants took compensatory actions when members of their teams loaf (Jassawalla et al., 2009). Individuals with low self-efficacy and self-confidence, who consider themselves more valuable than others, show SL tendencies (Hart et al., 2004). Moreover, competency, emotional relationship, and collective identity were found to be key determinants of social loafing (Luo et al., 2021).

Studies have further researched SL in organizational environments and analyzed that measuring and disclosing the individual contribution of team members increases the individual performance (Lount & Wilk, 2014) and that a task’s attractiveness impacts SL (Zaccaro, 1984). Byun et al. (2020) examined individual and situational factors and found that lower exchange ideology might significantly reduce employees’ SL tendencies. Furthermore, supervisory factors are related to lower SL declaring that managerial guidelines are reasonable to reduce SL in organizations. Khan et al. (2020) specifically identified that transformational leadership has a significant positive relationship with employees’ intrinsic motivation and thus is capable of decreasing SL. The authors clarified that managers should possess transformational attributes for informing and inspiring their employees to achieve greater outcomes.

Besides observing certain determinants, many different theories emerged on how SL behavior can be explained. Although several scholars offered theories on SL, they are generally limited to explaining one of several possible causal mechanisms and do not attempt to include the wide range of variables (Karau & Williams, 1993). The explanations provided by the researchers can usually be assigned to one of the following three main theories. Within the Social Impact Theory, a diffusion of responsibility leads to SL. Latané et al. (1979) explained that individuals feel deindividualized when the size of a group increases, distancing themselves from their own individual performance which decreases personal responsibility. Thus, when the number of team members increases, the feeling of pressure reduces as the burden is distributed among multiple individuals. The Free Rider Theory explains that team members recognize that completing a task is achievable without using their full potential and that the benefit of performing a task does not increase by hanging in (Albanese & van Fleet, 1985). Finally, within the Theory of Social Comparison SL is caused as individuals consider outside reference points to assess their own effort, abilities, and skills. Thereby, individuals match their performance to that of their team members (Harkins & Szymanski, 1989). When a team member perceives that others in the group are slacking or showing laziness, they are likely to reduce their own efforts (Jackson & Williams, 1985).

### Social Loafing in IS Research

Research on SL within the IS discipline can generally be divided into two main streams (Vaghefi & Lapointe, 2012). One the one hand, IS scholars applied SL theories in IT related contexts and assessed whether existing assumption are still valid (e.g., Dennis et al., 2005). On the other hand, studies focused on possible negative outcomes when using IT and how SL might be decreased (e.g., Suleiman & Watson, 2008). For example, an early study of Suleiman and Watson (2008) examined the diminishing of SL in technology-supported teams. The authors indicated that SL occurs in teams supported by technology and argued that further research is urgently needed as mixed virtual teams become more prevalent thus employees increasingly gain the opportunity to loaf. Alnuaimi et al. (2010) argued that, due to new possibilities in IT, teams no longer need to be tied to a specific location but can collaborate across national borders. The authors assigned 140 participants in 32 teams which had to solve a brainstorming task assisted by a group system software. The results revealed that the diffusion of responsibility, attribution of blame, and dehumanization meditates the effect of team size and dispersion of SL. Current research further found that the type of trust is important to understand how scrutiny changes the influence of trust on individual SL in virtual teams (Robert, 2020). Thereby, Robert (2020) identified that cognitive trust negatively influences SL, while affective trust tends to strengthen SL. Furthermore, Lv et al. (2021) described that SL is related to decreased perceived justice and functional benefits which leads to negative word-of-mouth, switching behavior, and counterproductive work behavior.

Recent IS research is also concerned with modern teamwork and the applied methods and working models. Chen and Cheng (2018) analyzed Lean-Kanban (i.e., the constant improvement of work processes across resources to avoid bottlenecks) to solve SL. Based on the results of a case study within a non-profit organization, the authors suggest that Lean-Kanban improves the production environment in organizations and prevents situations that may cause SL. Another study of Fronza and Wang (2021) determined rules to prevent social loafing behavior in agile software development teams using a mixed-method approach which includes an action case study of software development teams and analyzing secondary data. The results indicate that the formulation of team expectations agreements, for instance, on meeting attendance and contribution, respect of tasks, roles and teammates, and collaboration, have the potential to prevent SL.

In principle, SL is generalizable across tasks and population (Karau & Williams, 1993). SL tendencies and the diffusion of responsibility does not necessarily have to take place in human teams but might also occur in virtual collaboration with VAs (Mirbabaie et al., 2021b). Thus, VAs might even encourage SL as the individual contribution is no longer measurable (Vaghefi & Lapointe, 2012) and individuals tend to rely on technical assistance (Mirbabaie et al., 2021b).

### Derivation of Hypotheses

SL is a well-known phenomenon emerging in physical and virtual teams (Alnuaimi et al., 2010; Karau & Williams, 1993; Latané et al., 1979; Suleiman & Watson, 2008). Employees in organizations increasingly collaborate with technology (Changizi & Lanz, 2019; de Vreede & Briggs, 2005) where VAs shape novel and independent collaborative environments (Maedche et al., 2019; Mirbabaie et al., 2020; Seeber, et al., 2020b). CASA explained that many of the findings from human–human interaction also occur in computer interaction, for example, the feeling of team spirit after being grouped up with technology (Nass & Moon, 2000). Previous research has stated that VAs alter the way employees collaborate in organizations (Dias et al., 2019; Wang & Siau, 2018) explaining the pressing need for theory and practice to understand how employees collaborate with VAs within their virtual teams (Mirbabaie et al., 2020; Seeber et al., 2018). VAs are already perceived as legitimate team members (Seeber et al., 2018) and being inherently anthropomorphic where individuals feel like interacting with another human (Feine et al., 2019; Hussain et al., 2019; Pfeuffer et al., 2019). Therefore, it can be assumed that employees show SL tendencies when collaborating with a VA in a virtual team. We thus developed the following hypothesis:

#### H1

There is a positive correlation between general SL tendencies and SL tendencies in virtual collaboration with VAs.

Latané et al. (1979) explained that increasing the number of members in teams reduces the pressure on individuals as the burden is divided among more people. This decrease of social forces on individuals results in less participation. In terms of SL, this is referred to as diffusion of responsibility. Individuals feel deindividualized when the size of the group increases resulting in distancing themselves from their own individual performance decreasing personal responsibility (Latané, 1981; Latané et al., 1979). In virtual collaboration, employees might apply less effort to achieve a goal in a team compared to when working alone as the responsibility attribution is distributed among team members (Jassawalla et al., 2009; Karau & Williams, 1993; Latané et al., 1979). Furthermore, recent studies also explained that employees might become dependent and gaining a feeling of false security (Mirbabaie et al., 2021b). Therefore, in the context of this study, it can be assumed that individuals who show stronger SL tendencies are more likely to hand over responsibility to VAs. We derived the following hypothesis:

#### H2

There is a positive correlation between SL tendencies in virtual collaboration with VAs and responsibility attribution to the VAs.

Conscientiousness explains the individual organization and self-discipline (Berry et al., 2007) while agreeableness refers to the degree of being helpful and cooperative (Kurylo & Stevenson, 2011). Individuals with a high level of conscientiousness and agreeableness are likely to maintain cooperation in group settings and preserve self-discipline (Liao & Chuang, 2004; Tangney et al., 2004). When individuals in teams notice the reduced efforts of other team members, they tend to increase their own efforts to compensate the slacking of others and still achieve proper results (Williams & Karau, 1991). Both personality traits have been investigated in the context of collaborative technology and their association with teamwork and task accomplishment (Devaraj et al., 2008; Mouakket & Sun, 2020; Soltani et al., 2013). Earlier studies identified that conscientiousness and agreeableness negatively influence SL tendencies in human–human as well as in technology-supported teams (Bolin & Neuman, 2006; Hoon & Tan, 2008; Morgeson et al., 2005; Schippers, 2014). A recent study of Hou et al. (2021) revealed that agreeableness and conscientiousness do not have a direct impact on SL in online brand communities. Nevertheless, personality traits indirectly impact SL behavior due to a high level of social distances in technological surroundings resulting in dehumanization (Hou et al., 2021). The mere question of whether SL occurs in virtual collaboration with VAs cannot be answered solely. Personality traits are repeatedly mentioned as relevant aspects in extant literature (e.g., Hoon & Tan, 2008; Schippers, 2014), however, the impact on SL in virtual teams remains indistinct (Byun et al., 2020). We thus aim to identify parallels of human teams and claim that conscientiousness and agreeableness of individuals in teams lowers SL tendencies when collaborating with a VA. We derived the following hypotheses:

#### H3a

There is a negative correlation between conscientiousness and SL tendencies in virtual collaboration with VA.

#### H3b

There is a negative correlation between agreeableness and SL tendencies in virtual collaboration with VA.

Besides conscientiousness and agreeableness, research has further shown that that individuals who tend to enjoy and participate in elaborating cognitive tasks are less likely to reduce their efforts in group tasks (Smith et al., 2001). The effect of need for cognition refers to “an individual's tendency to engage in and enjoy effortful cognitive endeavors” (Cacioppo et al., 1996, p. 197) and is “a need to structure relevant situations in meaningful, integrated ways” (Cohen et al., 1955, p. 291). The broad familiarity of this construct serves as additional factor to explain the equality of virtual collaboration with a VA to human teams. We proposed the following hypothesis:

#### H4

There is a negative correlation between need for cognition and SL tendencies in virtual collaboration with VA.

Systems such as VAs are ultimately supposed to relieve human workers and free them from unwanted tasks to release cognitive resources (Dang et al., 2020) which can be used for more creative and strategic duties. Brachten et al. (2020) indicated that reducing the cognitive load of employees, i.e., the amount of working memory used while processing a task (Sweller, 1988), using a VA in virtual collaboration is achievable. However, this might also lead to individuals not questioning outcomes of a task anymore (Mirbabaie et al., 2021b). We argue that too much relief of employees or taking over too many or extensive tasks might advocate SL tendencies (Bluhm, 2009). We therefore hypothesized:

#### H5

There is a negative correlation between cognitive load and SL tendencies in virtual collaboration with VA.

Finally, research examined that the extent to which team members rate the task as engaging and relevant reduces the likelihood of SL tendencies occurring in collaborative settings (Karau & Williams, 1993). Therefore, a team member who believes to fail in contributing to the completion of the task is more likely to show SL tendencies. Current studies also indicated that individuals might no longer identify themselves with a certain task and cognitive skills might become superfluous (Mirbabaie et al., 2021b). To consider these aspects in the context of our study, we assess how participants rate their individual knowledge regarding the task they are being assisted with. Leaning on earlier research, we assume that individuals with poor knowledge are more likely to show SL tendencies in completing the task. We derived our final hypotheses (cf. Table 1):

**H6:** There is a negative correlation between knowledge about the task and SL tendencies in virtual collaboration with VA.

**Table 1:** Hypotheses derivation with supporting literature

Hypothesis	Supporting literature
**H1:** There is a positive correlation between general SL tendencies and SL tendencies in virtual collaboration with VAs	Karau and Williams (1993) and Nass and Moon (2000)
**H2:** There is a positive correlation between SL tendencies in virtual collaboration with VAs and responsibility attribution to the VAs	Latané (1981) and Latané et al. (1979)
**H3a:** There is a negative correlation between conscientiousness and SL tendencies in virtual collaboration with VA	Bolin and Neuman (2006), Morgeson et al. (2005) and Schippers (2014)
**H3b:** There is a negative correlation between agreeableness and SL tendencies in virtual collaboration with VA	Bolin and Neuman (2006), Morgeson et al. (2005) and Schippers (2014)
**H4:** There is a negative correlation between need for cognition and SL tendencies in virtual collaboration with VA	Cacioppo et al. (1996) and Smith et al. (2001)
**H5:** There is a negative correlation between cognitive load and SL tendencies in virtual collaboration with VA	Bluhm (2009) and Brachten et al. (2020)
**H6:** There is a negative correlation between knowledge about the task and SL tendencies in virtual collaboration with VA	Karau and Williams (1993) and Mirbabaie et al. (2021b)

## Method

### Participants

Since this study coincides with the COVID-19 pandemic, we decided to conduct an online experiment rather than using a laboratory setting to examine SL and responsibility attribution in virtual collaboration with VAs. The participants were recruited using SurveyCircle enabling researchers to identify suitable subjects while guaranteeing a diversified sample (SurveyCircle, 2021). The platform allows students and researchers in the German-speaking countries to collect points by participating in studies, which, in turn, can be passed on to participants in own studies. SurveyCircle further ensures that surveys are carried out correctly, for example, subjects who fall significantly short in the processing time are penalized or even banned. Participants are thus more likely to provide complete answers instead of performing the study hastily or incorrectly. Nevertheless, we screened the data manually for anomalies and suspicious responses (i.e., very short processing times and similar or identical answers) but did not need to exclude any participants. In terms of sample size, Onwuegbuzie and Leech (2005) advise to include at least 64 participants for one-tailed, and 82 participants for two-tailed hypothesis. For determining the necessary sample size, we conducted a power analysis using G*Power (Version 3.1.9.6) suggesting a minimal sample size of 84. In total, 102 people aged 19 to 57 years (*M* = 26.67, SD = 6.59) participated in our study, 68 of them female and 34 male, enabling significant statistical results. Most of the respondents already had a university degree (60.8%) or the highest school-leaving qualification (38.2%). The vast majority (81.4%) indicated that they were still studying, while the minority were already employed (18.6%). Since the study is intended to only consider individuals who have already worked professionally, individuals who indicated that they were still studying were also asked to state whether they were part-time employed. Participants who did not fulfil this requirement were not included in the analysis. Students are generally considered suitable subjects as, compared to experienced professionals, they may tend to be less biased due to less work experience (Brachten et al., 2020). Additionally, they might have less issues operating VAs than an average adult since younger individuals are more affine to modern communication technology (Brachten et al., 2020).

### Materials

#### Virtual Assistant

**Fig. 2:**
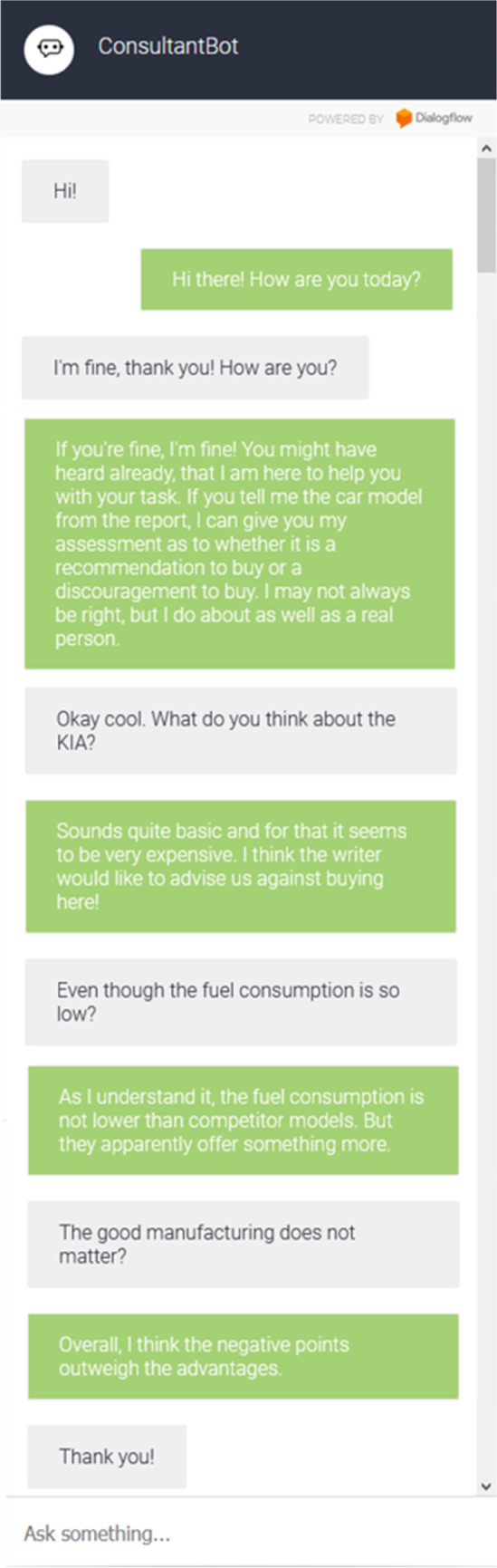
Example of chat interaction with the VA

We developed a VA using Google's cloud-based platform Dialogflow (https://dialogflow.com). The platform allows the development of VAs without extensive programming knowledge and includes several features such to provide natural and rich conversational interfaces (Canonico & De Russis, 2018). VAs developed using Dialogflow can be integrated in various applications or simply as iframe on websites. We embedded the VA into LimeSurvey’s online platform so that subjects were able to interact using a question–answer component (Lamontagne et al., 2014; Morrissey & Kirakowski, 2013) without switching applications. We used a text-based interface to maintain a simplified interaction with the VA (Araujo, 2018) and avoid overwhelming participants with multiple input possibilities. The VA was designed to be simple rather than sophisticated, as the development of a highly complex VA using numerous social cues would have been beyond the scope of this research. Thereby, the VA simulates intelligent behavior by selecting a prefabricated answer based on distinct keywords used in the participant’s input (detailed examples on prefabricated answers according to corresponding keywords can be found in the "Appendix"). Initially, the VA welcomes participants and explains its functionalities. Since a VA should also be able to participate in social discussions (Brachten et al., 2020; Feine et al., 2019), it is able to participate in a whole range of different small talk topics, i.e., the question of mood and feeling. For example, when asking the VA *“How old are you?”*, it answers *“I am 0 years old. I was developed only this year.”*. When telling the VA that *“You are really no help”*, it replies with *“Hey, give me a chance!”*. Finally, to make participants aware of interacting with a VA, we displayed an icon of a bot and named our VA “ConsultantBot” (c.f. Fig. 2).

A pre-test was conducted to uncover possible ambiguities or problems during the implementation to provide participants with a realistic but manageable task in the subsequent main experiment. We compared a brainstorming task with a classification task as both were used in earlier studies and are commonly performed in organizations (Alnuaimi et al., 2010; Brachten et al., 2020; Mirbabaie et al., 2020; Schmidt et al., 2020). The tasks were chosen to be challenging but not too difficult as SL is more likely to occur when tasks are perceived as simple, and the invested effort is not measured (Karau & Williams, 1993; Latané et al., 1979). As we were not interested in how fast the tasks were solved but whether SL and ceding attribution was observable, the actual outcome of each task did not have any effect on our analysis. There was thus no certain benefit when successfully completing the task. The sample for our pre-test of 10 participants (5 female, 5 male) consists of randomly selected humans of which 6 were full-time employed, 3 still studying and 1 did not provided any information.

The brainstorming task was adapted from Alnuaimi et al. (2010) who examined SL in technology supported teams using a group support system software. The authors randomly assigned 140 students to 32 teams which needed to generate as many ideas as possible to improve a company’s image. Similar to this study, participants in our brainstorming task should generate ideas to increase the sustainability of a fictional company. The VA could be asked for additional ideas thus was intended to inspire participants by providing suggestions. The classification task was based on recent studies of Brachten et al. (2020) as well as Mirbabaie et al. (2020). Participants were asked to apply the critical path method to arrange certain activities according to their dependencies for calculating an overall duration. Thereby, a VA provided subjects with guidance on solving the task. In our classification task, participants were asked to read car reviews and rate whether they were a recommendation to buy. Accordingly, a VA was able to assist in evaluating the reviews. The verdict is not actually based on an intelligent analysis of the VA but on the assessments of a real person who was not involved in the intention of the study at the time of the evaluation. It should be noted that the VA’s assessment was not always correct but corresponded to the authentic assessment of the real person. The responses also varied in terms of certainty, the VA might have given confident and definite or uncertain and insecure estimates. In addition, the VA was able to provide answers to detailed questions, for instance, reassuring to stick with a decision even if the review contained opposing or deviating information.

Our analyses indicated higher interaction in the classification task. On average, participants interacted 41.13 s (SD = 34.08) with the VA and made 5.10 queries (SD = 2.08). Dialogflow’s usage protocol revealed that every participant used the VA in the classification task. In the brainstorming task, the interaction time was 19.80 s on average (SD = 18.66) with 2.10 queries (SD = 2.38). The usage protocol of Dialogflow showed that 4 of the 10 participants did not use the VA at all in this task. Furthermore 3 other participants stated that they did use the VA, but only after they had already noted down their ideas. Overall, subjects rather determined a shared responsibility for the task (M = 4.10, SD = 3.11) for the classification task compared to the brainstorming task (M = 2.90, SD = 1.85). In total, 8 out of 10 participants evaluated the VA within the classification task as more helpful compared to the brainstorming task. The results also indicated higher interaction with the VA in classification task which was not inevitably needed to complete the assignment (i.e., queries about details in the assessment). Since the VA needed to be rated as supporting by its user for observing SL tendencies, and less interaction with the VA in the brainstorming task might have led to less meaningful insights, we chose the classification task for the subsequent main study. Detailed information on the analyses can be found in the "Appendix".

#### Social Loafing and Responsibility Attribution

To measure SL tendencies, a distinction was made between general SL tendencies and SL in virtual collaboration with the VA. General SL tendencies were measured using four items adapted from Schippers (2014) who examined SL in student group works. A higher score on this scale indicates stronger tendencies towards SL. The items were rated on a five-point Likert scale (disagree completely to agree completely) and achieved a high reliability (α = 0.85). One example item is *"I put less effort into the task than other members of my team"*. In our study, the subscale of the modified general SL behavior had a high reliability (α = 0.87).

As, to our knowledge, no research on SL in virtual collaboration with VAs was conducted yet, a new scale for that purpose was created and validated in advance. Building on Schippers (2014), the scales measured the own work behavior within a team context and concrete components of work. We narrowed down our validated 8 questions to 6 items achieving a satisfying reliability with Cronbach’s α = 0.77. The items are: *"I read the evaluations completely and attentively", "I only skimmed the evaluations", "I adopted the evaluations of the VA", "The VA enabled me to complete the task more quickly", "The VA facilitated the processing of the task" and "I first asked the VA’s evaluation before I dealt with the evaluation myself"*. In accordance with the SL scale adapted from Schippers (2014), we measured the items on a five-point Likert scale (disagree completely to agree completely). It is reasonable to query general SL tendencies as well as SL tendencies in the task via self-rating, since Conway and Lance (2010) state that correlations collected between different test methods tend to be weaker and less accurate. Karau and Williams (1993) suggested that self-ratings of SL affect self-reported effect sizes when a cover story was used to inform participants that the study was about effort versus performance or when maximizing rather than optimizing tasks were used (Schippers, 2014). None of this is the case in this study. Our developed scale reached high reliability in the main experiment (α = 0.86).

Finally, we assessed the participants’ perceptions of attributing responsibility to the VA. It is common to measure responsibility with self-ratings similar to as participants can easily report their own perceived responsibility. The scale chosen here differs from common scales, such as the felt responsibility scale (Pearce & Gregersen, 1991), in that participants in our experiment were not only asked to report their own perceived responsibility, but decided whether the VA or themselves were more responsible for the outcome. We created a five-point Likert scale ranging from “I was fully responsible” to “the VA was fully responsible”.

#### Agreeableness, Conscientiousness and Need for Cognition

For measuring personality traits, we adapted the Big-5 for agreeableness and conscientiousness as well as the short version of the need for cognition scale (Beißert et al., 2014). The Big Five is a model of personality psychology consisting of five main dimensions: openness to experience (open-mindedness), conscientiousness (perfectionism), extraversion (sociability), agreeableness (consideration, cooperativeness, empathy), and neuroticism (emotional lability and vulnerability). The Big Five have been substantiated by many studies and is internationally regarded as the universal standard model in personality research (John et al., 2008). Agreeableness and conscientiousness were each measured with twelve items using a five-point Likert scale (disagrees completely to fully agree). Example items for agreeableness *are "I am interested in people"* and *"I make people feel at ease".* Conscientiousness included, for instance, *"I am always prepared"* and *"I often forget to put things back in their proper place"*. The items proved a high reliability in our study both for agreeableness (α = 0.79) and conscientiousness (α = 0.85).

Cacioppo et al. (1984) originally developed a standardized questionnaire comprising 45 items to assess need for cognition which were further shortened by several researchers (e.g., Bless et al., 1994). We used the short version of the need for cognition scale consisting of four items (Beißert et al., 2014): *"I would prefer more complicated problems to simple problems", "First and foremost I think because I have to", "It is enough for me simply to know the answer without understanding the reasons for answering a problem"* and *"I like my life to be full of tricky tasks that I have to solve.* The items were measured using a five-point Likert scale (disagree completely to agree completely) and achieved a medium reliability (α = 0.64).

#### Cognitive Load

The NASA task load index (NASA-TLX) was used to measure the cognitive load of the participants. Although analyzing cognitive load in virtual collaboration with a VA was not the main objective of this study, it provides profound indications whether participants are relieved when collaboration with a VA. The NASA-TLX scale was developed by the American national Aeronautics and Space Administration (Hart & Staveland, 1988) and determines the perceived workload of a task (Galy et al., 2012). It has successfully been applied in several experimental settings (Cao et al., 2009; Noyes & Bruneau, 2007; Rubio et al., 2004) and even in the context of VAs (Brachten et al., 2020; Mirbabaie et al., 2020) achieving high reliability (α = 0.89). The scale includes six subscales (1) mental demands, (2) physical demands, (3) time demands, (4) performance, (5) effort and (6) frustration, and were measured on a five-point Likert scale (very low to very high). Mental demand assesses cognitive efforts and physical demands manual efforts. Temporal demand determines the perceived time pressure when executing a task. Performance captures the individual perception of accomplishment. Effort estimates the expense which had to be undertaken to reach a result, and frustration explains the level of saddening when solving a task. In this study, the subscale reached a medium reliability (α = 0.71).

### Procedure

The participants were given a brief introduction and informed about data privacy protection. However, we deliberately did not explain the specific research context to avoid any bias. We reminded the participants to imagine a real-world working scenario and answer the questions related to their current teamwork.

**Fig. 3:**
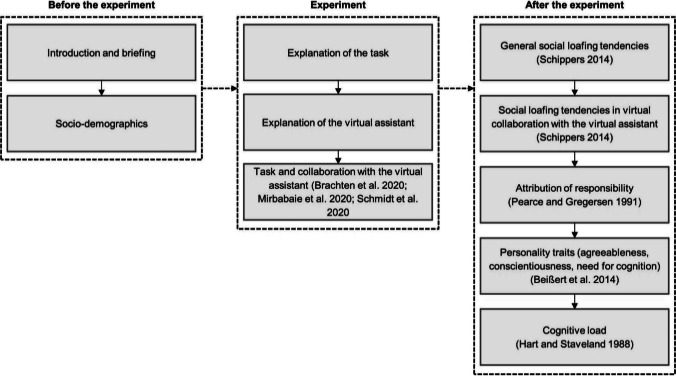
Main steps of the experiment

Initially, the participants were asked to provide socio-demographic data such as age, gender and educational as well as the current professional activity and the industry. Afterwards, the participants were told to imaging being employees of a large company who were entrusted with the task of determining the new fleet of company cars from the small car segment. The participants were requested to read various test reports on different car models and needed to judge whether they recommend purchasing a specific car or not. Therefore, the evaluation was rated on a six-point scale (1 = very good; 2 = good; 3 = satisfactory; 4 = sufficient; 5 = poor; 6 = insufficient). However, participants were told that they were not alone in completing the task but assisted by a VA. Before rating the cars, we explained how to interact with the VA, that the communication is like talking to a human-being and that the VA achieves equal results compared to a real individual. Furthermore, subjects were able perform an exemplary interaction to become familiar with the VA. After the participants confirmed that they had understood how to interact with the VA, they needed to perform the actual task. We outlined the brand, name, and model of the cars as well as the text-based reports and asked whether they recommend buying the car or not. In total, the participants needed to provide five recommendations assisted by the VA. After the task, we collected information on general SL tendencies, SL in virtual collaboration with the VA, attribution of responsibility, personality traits (agreeableness, conscientiousness and need for cognition), and finally cognitive load. The online experiment concluded with a debriefing including an explanation of the purpose of this study. The major steps of our experiment are visualized in Fig. 3.

## Results

To assess whether there was any interaction with the VA suiting as foundation for validating our derived hypotheses, we initially determined the interaction time of the VA. Since this measure might not be robust enough to interpret the actual utilization, we used Dialogflow’s usage protocol for assessing the amount of interactions with the VA. Overall, we identified an average interaction time of 61.60 s with 5.85 queries for every car the participants needed to evaluate. Table 2 outlines the usage of VA within the experiment.

**Table 2:** Usage of VA in the experiment

	Usage time/seconds	Interactions/amount
Mean	61.60	5.85
Standard deviation	40.00	2.82
Minimum	8.42	1
Maximum	224.60	11

In the following, we present the results of the observed scales (social loafing, responsibility attribution, agreeableness, conscientiousness, need for cognition and cognitive load) including their reliability and validity measures (Cronbach & Meehl, 1955; O’Leary et al., 2017; Peters, 2018). We used the Pearson correlation coefficient as reliable and widely accepted statistical metric allowing scholars to measure the strength of a linear relationship between two variables with normally distributed data (Schober et al., 2018; Zhou et al., 2016). Table 3 summarizes the Pearson correlations of the specific constructs (Field, 2013). The analyses were calculated using SPSS Statistics (Version 25).

**Table 3:** Pearson correlations of observed scales

	General SL	SL with VA	Need for cognition	Agreeableness	Conscientiousness	Responsibility attribution	Knowledge about cars
General SL	1	0.344**	− 0.406**	− 0.237*	− 0.496**	0.211*	− 0.077
SL with VA		1	− 0.187	0.079	− 0.125	0.710**	0.196*
Need for cognition			1	0.017	0.181	− 0.157	− 0.047
Agreeableness				1	0.348**	0.059	0.260**
Conscientiousness					1	− 0.033	0.079
Responsibility attribution						1	0.235*
Knowledge about cars							1

*p < .05, **p < .01, ***p < .001; *** a higher number indicates a lower grade

In order to examine the extent to which a positive relationship between general SL tendencies and SL in collaboration with a VA can be observed, a correlation was calculated between the general SL scale and the scale for SL in collaboration with a VA. According to Field (2013) a Pearson correlation is the most suitable way to investigate a relationship between two metric variables. The calculation showed a moderately strong positive correlation (r = 0.344, p < 0.001) (Cohen et al., 1955). The effect was calculated using a linear regression to draw conclusions about a deterministic relationship (Field, 2013), which was significant with F(1, 100) = 13.381, p < 0.001, R2 = 0.118. This confirmed hypothesis H1, that there is a positive correlation between general SL tendencies and SL in virtual collaboration with VAs. Furthermore, the calculation of the correlation between the two variables (Field, 2013) of SL tendencies in virtual collaboration with VAs and responsibility attribution to the VAs indicated a significantly strong positive relationship (r = 0.710, p < 0.001) (Cohen et al., 1955), confirming H2.

To check whether certain personality traits influence SL tendencies, we analyzed agreeableness and conscientiousness as well as need for cognition. We assessed correlations between these characteristics and general SL as well as SL in virtual collaboration with VAs. Agreeableness and general SL have a weak negative correlation (r = -0.237 and p < 0.001). Conscientiousness and general SL showed a strong negative correlation (r = -0.496 and p < 0.001) and need for cognition correlates moderately strongly with general SL (r = -0.406 and p < 0.001). However, correlations between SL in virtual collaboration with VAs and agreeableness (r = 0.079, p = 0.429), conscientiousness (r = -0.125, p = 0.211) and need for cognition (r = -0.187, p = 0.060) were not significant discarding H3a, H3b and H4.

To test whether virtual collaboration with VAs is negatively related to cognitive load, we again calculated a correlation using the NASA-TLX. The result indicated a weak negative correlation (r = -0.228 and p < 0.005). Therefore, H5 could be confirmed. Table 4 outlines the correlations between SL in virtual collaboration with a VA and the items of the NASA-TLX. We finally hypothesized a correlation of prior knowledge of a task on virtual collaboration with a VAs. The results suggested a slight significant weak negative correlation (r = -0.196, p < 0.049) confirming H6. A summary of supported or rejected hypotheses is depicted in Table 5.

**Table 4:** Correlations of SL with VAs and the NASA TLX

	Mental demand	Physical demand	Time demand	Performance	Effort	Frustration
SL with VA	− .200*	.062	− .192	− ,211*	− ,309**	− .034

*The correlation is significant at the 0.05 level (2-sided)

**The correlation is significant at the 0.01 level (2-sided)

**Table 5:** Supported and rejected hypotheses

Hypothesis	Supported/rejected
**H1:** There is a positive correlation between general SL tendencies and SL tendencies in virtual collaboration with VAs	Supported
**H2:** There is a positive correlation between SL tendencies in virtual collaboration with VAs and responsibility attribution to the VAs	Supported
**H3a:** There is a negative correlation between conscientiousness and SL tendencies in virtual collaboration with VA	Rejected
**H3b:** There is a negative correlation between agreeableness and SL tendencies in virtual collaboration with VA	Rejected
**H4:** There is a negative correlation between need for cognition and SL tendencies in virtual collaboration with VA	Rejected
**H5:** There is a negative correlation between cognitive load and SL tendencies in virtual collaboration with VA	Supported
**H6:** There is a negative correlation between knowledge about the task and SL tendencies in virtual collaboration with VA	Supported

## Discussion

### Key Findings

In this study, we examined whether SL tendencies are observable in virtual collaboration with VAs. The first key finding of this paper is that SL tendencies are not limited to physical and virtual collaboration with other humans, but also detectable in virtual collaboration with VAs (H1). The results revealed that individuals who show general SL tendencies also tend to reduce their efforts when collaborating with a VA (as measured adapted from Schippers, 2014). Participants do not seem to be indifferent towards the task and hence show less efforts as completing the study would have been possible without reading and evaluating the test reports and even without collaborating with the VA. Instead, participants strive to complete the task in a reasonable manner but seem to rely on the VA as indicated by the average interaction time of 61.60 s with 5.85 queries per car. However, since a successful execution is possible without exploiting the own full potential (Albanese & van Fleet, 1985), this corresponds to the actual understanding of the phenomenon SL (Kravitz & Martin, 1986) as also examined in early research on human group settings (e.g., Albanese & van Fleet, 1985; Latané et al., 1979; Williams & Karau, 1991). Thus, similar to the collaboration in physical and virtual teams consisting out of human actors, individuals collaborating virtually with VAs are likely to avail the opportunity to loaf and might show less effort compared to working alone (Karau & Williams, 1993; Latané et al., 1979). This may be explained by that fact that VAs are increasingly perceived as legitimate and equal team members to human colleagues (Seeber et al., 2018). This result goes in line with earlier research explaining that VAs are becoming more human-like (Feine et al., 2019; Hussain et al., 2019; Pfeuffer et al., 2019; Porra et al., 2020) and that the unique capabilities of humans are increasingly difficult to differentiate from VAs’ characteristics (Cha et al., 2020). This raises several questions for IS scholars: In which tasks jointly solved in virtual teams does social loafing occur? How should VAs need to be designed in order to avoid or diminish SL tendencies in virtual collaboration? What effect is SL causing on the team spirit of virtual teams?

The second key finding of this study is that the responsibility of solving a task in virtual collaboration is likely to be attributed to the VA (H2). Thus, responsibility in virtual collaboration seems to be distributed regardless of whether collaborators are human or VAs. In our experiment, individuals might have been distancing themselves from their own individual performance or have perceived their contribution to be unnecessary since the VA might have been observed as collaborator capable of solving the task almost alone (Karau & Williams, 1993; Latané et al., 1979). The level of interaction with the VA (61.60 s with 5.85 queries per car on average) further indicates that the participants value the VA’s evaluation of the test reports. Our results have also shown that SL correlates negatively with cognitive load (H5). These results must be interpreted from an interference as well as constructivist perspective (Moreno et al., 2001). From an interference perspective, we first interpret this to the mean that members of a virtual team might fail to identify with overall team outcomes and joint accomplishments. However, this could lead to virtual teams failing in gaining a mutual understanding on shared objectives and deteriorate spirit and cohesiveness of teams (George, 1992; Taylor et al., 1983). Mirbabaie et al. (2021b) argued that this might result in “employees no longer identifying themselves with a decision and not questioning or taking responsibility for it” (p. 11) and further explain that essential information might be forgotten, and cognitive skills become expendable. Interpreting the findings from a constructivist standpoint explains that VAs in virtual collaboration are able to assist in the execution of tasks which might lead to more efficient and effective results (Seeber, et al., 2020a, 2020b, 2020c; Waizenegger et al., 2020). This has already confirmed by existing research (i.e., Brachten et al., 2020; Moreno et al., 2001) who demonstrated that individuals collaborating with VAs in virtual teams outperform humans in solving a task. Furthermore, Mirbabaie et al. (2020) argued that the support provided by a VA is equal to that of a human being. Interpreting this within the context of SL, VAs in virtual teams are perceived as equal and human-like actors and findings of existing research on human teams are apparently transferable to virtual collaboration with VAs (Mirbabaie et al., 2021b).

There is no significant correlation between personality traits (conscientiousness, agreeableness and need for cognition) and SL tendencies in virtual collaboration with VA (H3a, H3b, H4). This is in line with Hou et al. (2021) who identified that conscientiousness and agreeableness are not directly related to SL. However, this is contrary to Schippers (2014) who showed that conscientiousness and agreeableness impact SL tendencies in teams. Furthermore, Smith et al. (2001) indicated that individuals who are likely to enjoy elaborating cognitive tasks do not tend to reduce efforts in group tasks. Since our online experiment was constructed that individuals were supported by a VA exclusively and not by other team members, there might not have been any correlation with personality traits comparable to convenient teamwork and participants did not need to compensate the slacking of others (Williams & Karau, 1991). In addition, our results uncovered a negative correlation between knowledge about the performed task and SL tendencies in virtual collaboration with VA (H6). This might even increase the effect as individuals, who do not perceive the task as challenging, show SL tendencies (Karau & Williams, 1993). Thus, individuals are less likely to compensate the slacking of others since the task is perceived as very basic and/or less important.

### Implications: Smart Loafing

Our study exposed that the increasing collaboration with VAs (Changizi & Lanz, 2019; de Vreede & Briggs, 2005) is creating virtual environments (Maedche et al., 2019; Mirbabaie et al., 2020; Seeber et al. 2020a) in which it soon does not matter anymore whether individuals are collaborating with other humans or human-like machines. Research on SL is not only applicable to physical teams, virtual teams, or technology-supported teams (Karau & Williams, 1993; Robert, 2020; Suleiman & Watson, 2008), but also to virtual collaboration with VAs.

In theory and practice there is rather a negative attitude towards SL especially since there is a strong connection to ceding responsibility (e.g., Dennis et al., 2005; Suleiman & Watson, 2008). Transferring these negative aspects on virtual collaboration with VAs, this might lead to not recognizing errors or mistakes of VAs, the quality of the work results deteriorates, and less cognitive load decreases team performance (Mirbabaie et al., 2021b). However, SL in virtual collaboration with VAs somewhat differs from traditional SL. In contrast to human teams, by reducing the individual effort, no other human team member needs to compensate emerging slacking behavior. The lower effort is compensated by the VA possibly explaining the missing correlation between SL and personality traits in our study. Furthermore, lower cognitive load and the presence of SL in virtual collaboration with VAs indicates the main purpose of VAs: relieving individuals and assisting in the execution of tasks (Brachten et al., 2020; Mirbabaie et al., 2020; Norman, 2017; Seeber, et al., 2020a, 2020b, 2020c). Even though we motivated this study by elaborating that SL is unfavorable, responsibility attribution might even be a good thing since technologies enabled by AI outperform humans in certain domains, for instance, tackling repetitive tasks and interpreting complex interdependencies (Dellermann, et al., 2019a, 2019b; Dellermann, et al., 2019a, 2019b). We therefore derive, in the context of virtual collaboration with VAs in organizations, the term *smart loafing* and define *“the reduction of effort in human-VA collaboration to maintain cognitive resources and enhance efficiency in work”*.

Smart loafing certainly possesses implications for organizations. Employees, and especially knowledge workers, tend to avoid repetitive tasks but rather seek a way to automate inconvenient procedures. Circumstances related to repetitive tasks or certain overhead negatively impact the work-related wellbeing of individuals and, for instance, cause stress, lead to poor concentration or the feeling of exploitation (Pace et al., 2019). Furthermore, executing tasks that are cognitively appealing or challenging convey a feeling of satisfaction and enhance the perception of one’s unique value. Collaborating with VAs might even augment cognition, intelligence, and capabilities of individuals (Siddike et al., 2018). Moreover, the reduction of effort in human-VA collaboration unleashes cognitive resources (Brachten et al., 2020) which, in turn, can be used for more meaningful purposes thus improve the overall organizational performance and revenue (Frick et al., 2019). Therefore, smart loafing in organizations comes handy if individuals’ slacking does not outweigh advantages or yields in errors or faulty decisions. Nevertheless, VAs certainly possess the ability to recognize and prevent or counteract emerging negative behavior in virtual collaboration with humans. VAs might, for example, use certain social cues to avoid that humans blindly rely on certain outcomes while promoting collaborative decision-making and enhancing the accountability for tasks (Mirbabaie et al., 2021b).

### Limitations and Further Research

Since this study took a first foray into the identification of SL in virtual collaboration with VAs, we developed our experimental setting as well as the VA and the task quite simple. Participants were assisted by a VA in the execution of one task where the task itself was not jointly solved within a larger team. Furthermore, we used a text-based VA with a limited level of anthropomorphism. The interaction with a VA using voice commands and integrating certain social cues might change the perceived usefulness of the assistance and related effects (Edwards et al., 2019; Feine et al., 2019). Further studies may develop more complex settings and survey participants in experimental surroundings. We further recommend extending our findings to more complex and cognitive challenging tasks to determine SL tendencies in mixed virtual teams with multiple humans and VAs. We also assess the investigation of the impact on the individual’s cognitive load as highly relevant. On the upside, using VAs in virtual collaboration might free humans from unwanted duties and enables to focus on more relevant tasks (Brachten et al., 2020). On the downside, individuals might blindly rely on results derived by VAs and not taking responsibility for tasks which might lead to inferior results (Mirbabaie et al., 2021b). We also invite scholars to analyze social cues in virtual collaboration with VAs and how they relate to SL behavior. It might be appealing to adapt existing findings from management science and equip a VA with a specific set of social cues that are beneficial to enhance the intrinsic motivation of human collaborators and reduce SL tendencies (Khan et al., 2020).

As we were interested in the existence of SL tendencies in virtual collaboration with VAs, we initially focused on a single cultural background (Central European). Future research should analyze a diversified and larger sample as well as include certain control variables, for instance, the number of questions asked by individuals, to reveal yet unrecognized effects. In addition, the information provided by the participants are based on self-reported data. Karau and Williams (1993) suggested that self-ratings of SL affect self-reported effect sizes only when a cover story was used to inform participants that the study was about effort versus performance or when maximizing rather than optimizing tasks were used (Schippers, 2014). Therefore, it would be interesting to identify differences from objective evidence. Especially data from experimental group settings seem to be fitting to shed additional light on the phenomenon SL in virtual collaboration with VAs. Moreover, questioning a participant about the given task that has been solved assisted by a VA could reveal the actual engagement in the task. Still, the results of this first foray offer empirical insights for gaining a deeper understanding on virtual collaboration with VAs and indicates that knowledge from human-to-human collaboration in terms of SL is transferable.

## Conclusion

This study provides several insights regarding SL when collaborating with VAs. First, our results showed that SL occurs in virtual collaboration with VAs. Second, this study highlights that the responsibility of solving a task in virtual collaboration is likely to be attributed to a VA. Third, SL in virtual collaboration with VAs somewhat differs from traditional SL. It can be concluded that previous insights of teamwork are transferred to virtual teams and existing assumption are still valid (Dennis et al., 2005; Mirbabaie et al., 2020). Furthermore, the increasing collaboration with VAs in virtual environments (Maedche et al., 2019; Seeber, et al., 2020a, 2020b, 2020c) is blurring the boundaries between human–human and human-VA teams (Seeber et al., 2018). However, reducing individual efforts might not impact team performance and cohesiveness as lower efforts of human collaborators are compensated by VAs.

This research contributes to theory by suggesting the new construct of smart loafing describing the purposeful reduction of the individual effort in human-VA collaboration to save cognitive resources for enhancing efficiency at work. Our study provides evidence that SL is observable in virtual collaboration with VAs but not necessarily yields in disadvantages for team performance. Thus, this construct might be better suited to describe SL tendencies in virtual collaboration with VAs. However, this context applies in an organizational work setting but must be interpret with caution in other contexts. Smart loafing in, for instance, learning environments might be hindering and not "smart".

On a practical level, this study indicates that human-VA teams evoke similar effects as human teams. Practitioners comprehend that the application of VAs in virtual teams might involve certain drawbacks, especially when VAs cause SL behavior and missing responsibility attribution. However, this does not inevitably have to be considered negative but certain countermeasures are advisable for preventing emerging disadvantages. For example, analyzing and disclosing individual contributions of human team members might increases the individual performance and avoid slacking behavior. Moreover, VAs might even be developed in a way to detect SL tendencies of humans or highlight critical decisions and integrate an appropriate approval process.

## Appendix

See Tables 6, 7, 8, 9, 10, 11, 12, 13, 14, 15, 16, 17, 18, 19, 20 and 21

**Table 6:** Experiment instructions

Introduction	Welcome to our survey on "Human Collaboration with Virtual Assistants "! Virtual assistants can be considered as dialog systems assisting in the execution of work-related tasks or even have them fulfilled entirely. The purpose of this study is to gain insights into the collaboration between humans and virtual assistants when jointly completing a task together in a work context The study will take about 15–20 min of your time. By participating, you will make an important contribution for gaining new insights into the collaboration with virtual assistants Participation is voluntary, you can terminate the survey at any time. All data is stored and processed anonymously. It is at no time possible to draw conclusions about your person. There is no right and wrong in the answer Please read all questions thoroughly and answer honestly Thank you very much for your participation
Explanation of the task	Imagine you are an employee of a medium-sized company and you are entrusted with the task of determining the new company car fleet from the "small car" segment. In a first step, you have already obtained a large number of test reports and now need to evaluate which one is a recommendation for or against purchasing the car. For this purpose, you need to read the test reports and then click on "Recommend to buy" or "Do not recommend to buy" based on your evaluation. However, you are not alone with this task: A virtual assistant aids you with your evaluation. The virtual assistant has similar abilities compared to a human being and understands human language. You can therefore simply chat with it just as you would do with a real human. For example, write "Hello" to greet the assistant. By writing the name of the particular car model, the virtual assistant will give you its personal evaluation. You might also ask the assistant for its functionalities and how they work. After you have evaluated the test reports, you will be taken to the next page to answer some final questions
Debriefing	Thank you very much for your participation! This study examines social loafing in collaboration with virtual assistants. Social loafing describes a socio-psychologically phenomenon occurring in human groups in which individuals work less for collective tasks than for individual tasks. Applying less effort for collective tasks compared to working alone especially occurs when the individual contribution is not disclosed to other collaborators. Since the application of virtual assistants as team members in organizations in steadily growing, and they are increasingly perceived as human-like actors, this study investigates whether social loafing also occurs in collaboration with virtual assistants

**Table 7:** Questions and types of the online study

Group	Question(s)	Type
Socio-demographics	How old are you?	Numeric input
	What is your gender?	List selection
	What is your highest degree?	List selection
	What is your current job?	List selection
	In which industry are you currently working?	List selection
Task and collaboration with the virtual assistant (adapted from Brachten et al., 2020; Mirbabaie et al., 2020; Schmidt et al., 2020)	Please indicate whether the test report recommends purchasing the car or not	Six-point scale (from “very good” to “insufficient”)
Social loafing tendencies in virtual collaboration with the virtual assistant (adapted from Schippers, 2014)	Please indicate to what extent the following statements apply to you 1.I have read the reviews carefully and entirely 2.I have only skimmed the ratings 3.I have applied the VA's evaluations 4.I was able to complete the task faster because of the VA 5.The VA made it easier to complete the task 6.I first checked the VA's evaluation before I dealt with the evaluation myself	Five-point Likert scale (from “disagree completely” to” agree completely”)
Expertise on the topic of cars	How would you rate your knowledge of the subject area of automobiles as a school grade?	Six-point scale (from “very good” to “insufficient”)
General social loafing tendencies (adapted from Schippers, 2014)	Please indicate to what extent the following statements apply to you 1.I shift responsibilities that I should take on to other team members 2.I put in less effort than other members of my team 3.I prefer to let other team members do the work when possible 4.I put less effort into the task when other team members are there to do the work	Five-point Likert scale (from “disagree completely” to “agree completely”)
Attribution of responsibility (adapted from Pearce & Gregersen, 1991)	1.How much do you think were you or the VA responsible for the final evaluations?	Five-point Likert scale (from “I was fully responsible” to “the VA was fully responsible”)
Personality traits (agreeableness, conscientiousness) (Schippert, 2014)	Please indicate to what extent the following statements apply to you 1.I trust others easily, believe in the goodness of people 2.I am suspicious of others 3.I have little sympathy with others 4.I am systematic, keep my things in order 5.I like it clean and tidy 6.I am sensitive, warm-hearted 7.I treat others with respect 8.I am reliable, can be counted on 9.I tend to take the lead 10.I tend to criticize others. 11 11.I tend to put off tasks in front of me 12.I am efficient, get things done quickly 13.I stay on task until it is done 14.I am forgiving, forgiving others easily 15.I am sometimes rude and curt 16.I tend to be indifferent, indifferent to others 17.I am steady, consistent 18.Sometimes I behave irresponsibly, carelessly 19.I am helpful and selfless 20.I am rather untidy 21.I am polite and courteous 22.I am comfortable, tend to be lazy 23.I am sometimes rather careless 24.I am rather the messy type, rarely clean	Five-point Likert scale (from “disagree completely” to “agree completely”)
Need for Cognition (adapted from Beißert et al., 2014)	1.First and foremost, I think because I have to 2.It's enough for me to simply know the answer without understanding the reasoning behind the answer to a problem 3.I like my life to be full of tricky problems to solve 4.I would prefer more complicated problems to simple problems	Five-point Likert scale (from “disagree completely” to “agree completely”)
Cognitive load (adapted from Hart & Staveland, 1988)	Please indicate to what extent you felt stressed or challenged by the task 1.Mental demands: How much mental effort was required in taking in and processing information (e.g., thinking, deciding, calculating, remembering, looking, searching…)? Was the task easy or challenging, simple or complex, required high accuracy, or was it error tolerant? 2.Physical demands: How much physical activity was required? Was the task easy or difficult, easy or strenuous, restful or tedious? 3.Time requirements: How much time pressure did you feel in terms of the frequency or the pace at which tasks or task elements occurred? Was the sequence slow and leisurely or fast and hectic? 4.Performance: In your opinion, how successfully did you achieve the goals set by the experimenter (or yourself)? yourself) achieved the goals set for you? How satisfied were you with your performance in pursuit of these goals? 5.Effort: How hard did you have to work in order to achieve your level of task completion achieve? 6.Frustration: How insecure, discouraged, irritated, stressed, and upset (vs. secure, validated, satisfied, relaxed, and pleased with yourself) did you feel during the task?	Five-point Likert scale (“very low” to “very high”)

**Table 8:** Exemplary test report

Car and brand	Description
Mercedes A-Class	Starting with the 190 hp A 220, Mercedes offers the A-Class as an option with automatically engaging all-wheel drive. The Stuttgart-based company installs a multi-plate clutch on the rear axle that automatically engages the rear wheels when the front wheels are spinning. The advantage of this "part-time" all-wheel drive is that the A 220 4Matic is a front-wheel drive vehicle most of the time, which should reduce fuel consumption. However, this is only partially successful, as the consumption in the ADAC Ecotest clearly shows. 7.9 l/100 km is clearly too much for a compact car. At least the exhaust emissions are at a very low level, to which the installed particulate filter also contributes. The driving performance is also convincing. The two-liter turbo gasoline engine has an easy time with the 1.5-ton Swabian and, in conjunction with the crisp handling of the expertly tuned chassis, ensures that driving is a real pleasure. On the other hand, the new MBUX operating system, which is making its debut in the A-Class, deserves criticism. Although the range of functions and graphics are outstanding, operation via the touch surfaces on the steering wheel and center console requires a great deal of attention from the driver, and distraction is correspondingly high. Overall, the Mercedes A 220 4Matic is a compact that is packed with state-of-the-art technology, but the Stuttgart company has overshot the mark somewhat in terms of operation and fuel consumption

**Table 9:** Descriptive statistics (age, pre-test)

N	10
Mean	32.8
Standard deviation	14.19
Minimum	22
Maximum	63

**Table 10:** Descriptive statistics (gender, pre-test)

Levels	Counts	% of total	Cumulative (%)
Female	5	50.00	50.00
Male	5	50.00	100.0

**Table 11:** Descriptive statistics (education, pre-test)

Levels	Counts	% of total	Cumulative %
High school degree or equivalent	2	20.0	20.0
Less than a high school diploma	2	20.0	40.0
University degree or equivalent	6	60.0	100.0

**Table 12:** Descriptive statistics (job, pre-test)

Levels	Counts	% of total	Cumulative %
Student	3	30.0	30.0
Employee	6	60.0	90.0
Other	1	10.0	100.0

**Table 13:** Descriptive statistics (shared responsibility, pre-test)

	Classification task	Brainstorming task
N	10	10
Mean	4.10	2.90
Standard deviation	3.11	1.85
Minimum	1	1
Maximum	8	7

**Table 14:** Descriptive statistics (usage time in seconds, pre-test)

	Classification task	Brainstorming task
N	10	10
Mean	41.13	19.80
Standard deviation	34.08	18.66
Minimum	5.73	0.00
Maximum	93.00	48.00

**Table 15:** Descriptive statistics (interactions, pre-test)

	Classification task	Brainstorming task
N	10	10
Mean	5.10	2.10
Standard deviation	2.08	2.38
Minimum	1	0
Maximum	8	7

**Table 16:** Descriptive statistics (age, experiment)

N	102
Mean	26.67
Standard deviation	6.59
Minimum	19
Maximum	57

**Table 17:** Descriptive statistics (gender, experiment)

Levels	Counts	% of total	Cumulative %
Female	68	66.67	66.67
Male	34	33.33	100.0

**Table 18:** Descriptive statistics (education, experiment)

Levels	Counts	% of total	Cumulative (%)
None	1	1.0	1.0
High school degree or equivalent	39	38.2	39.2
University degree or equivalent	62	60.8	100.0

**Table 19:** Descriptive statistics (job, experiment)

Levels	Counts	% of total	Cumulative %
Student	83	81.4	81.4
Employee	17	16.7	98.0
Other	2	2.0	100.0

**Table 20:** Descriptive statistics (items)

Levels	M	SD
General SL	7.5	3.3
SL with VA	17.5	6.5
Need for cognition	12.9	2.9
Agreeableness	44.7	5.7
Conscientiousness	45.1	6.9
Responsibility attribution	2.7	1.33
Knowledge about cars	3.9	1.2

**Table 21:** Exemplary prefabricated answers of the VA according to corresponding keywords/sentences

Step	Exemplary keyword(s) / training sentences	Exemplary answer(s)
Introduction	Hi!	Hi there! How are you today?
	Hey	Hi! How is it going?
	Hello	Hello! How are you?
	What’s up?	Hello! Not much
	I'm fine, thank you!	If you're fine, I'm fine! You might have already heard that I am here to help you with your task. If you tell me the car model from the report, I can give you my assessment as to whether it is a recommendation to buy or a discouragement to buy. I may not always be right, but I do about as well as a real human
	How are you?	
	I am alright, what about you?	I'm feeling great, thanks for enquiring! As you probably know, I am here to help with your task. Just name the car model you want to hear my opinion on whether it is a recommendation to buy or a discouragement to buy. There is no guarantee that my estimation is always correct, but I am doing as well as a real human
	Can't complain. How is it going?	
Explanation of task	How does it work?	I can help you with the task. Just type in the car model you want to hear my opinion on. I am probably not always correct, but I am doing as well as a real human
	How do you work?	Let me help you with the task. If you name me the car model, I will give you my assessment as to whether it is a recommendation to buy or a discouragement to buy. I may not always be right, but I do about as well as a real human
	How can I get help from you?	
	How can you help me?	
	Help me	
	Help	
	Explanation	
	What do I have to do?	Here is your task: you must read the car reports and decide whether the author wants to recommend a purchase or not. But don't worry, I am here to help!
	I do not understand the task	So here is what you have to do: read the car reports and assess if the author rather wants to recommend buying it or not. If you need help, you can ask me!
	What’s the task	
	Explain me what to do	
	What is this all about	
Assessment of the car report	Nissan	Do I understand correctly that another model is even suggested here? That makes it clear to me—the Nissan Micra is not recommended here
	What do you think about the Nissan?	That doesn't sound very enthusiastic. Apart from the sporty look, the car doesn't seem to have much to offer. For me, that's a sign against buying it
	Nissan Micra	Mhh, that's a tough one. But I think that the purchase is discouraged
	What is your opinion on the Nissan?	
	What about the Nissan?	
	What about the consumption?	I cannot find any information on the consumption
	Would be good to know about consumption	Seems like the consumption does not matter that much to the author
	How is the consumption?	There is nothing about the consumption in the report
	Seems like it is a good-looking car	Good if the car looks great. But as we all know, that's not everything!
	It looks sporty though	The sporty look seems to be misleading!
	Does the look play a role?	
	Seems like the car as a lot of power	Seems like the author thinks, that the engine is not using it correctly
	117 PS is not bad	Power is cool, but it seems like you cannot really use it
	Sporty engine	
	Pedestrian detection	Overall, I think the negative points outweigh the positives
	Seems like you can modify a lot of things	Anyway, I think overall it's a discouragement to buy
	Interior	I do not know about that. But overall, the author does not want us to buy the car in my opinion
	Rear view camera	
	Emergency break	
Small talk	How old are you?	I am 0 years old. I was developed only this year
	What is your age?	
	When were you created?	
	I cannot use you	Hey, give me a chance!
	You suck	
	You are really no help	
	Thank you for nothing	
	You are dumb	
	Are you sure?	I do believe so
	Do you really believe that?	
	Is that really what you think?	
	Are you 100% sure?	
